# Micropeptides encoded by lncRNAs associated with cancer progression reveal novel immunogenic epitopes

**DOI:** 10.1093/bioinformatics/btag452

**Published:** 2026-08-21

**Authors:** Stav Zok, Michal Linial

**Affiliations:** The Rachel and Selim Benin School of Computer Science and Engineering, The Hebrew University of Jerusalem, Jerusalem, Israel; Department of Biological Chemistry, The Life Science Institute, The Hebrew University of Jerusalem, Jerusalem, Israel

## Abstract

**Motivation:**

Long non-coding RNAs (lncRNAs) regulate gene expression, chromatin organization, and cellular signaling. Recent studies indicate that **∼**20% of the **∼**36 000 human lncRNA genes harbor small open reading frames (sORFs) capable of producing micropeptides (MPs), whose functions remain largely unknown. Whether these peptides contribute to the cancer immunopeptidome is largely unexplored.

**Results:**

We systematically analyzed lncRNAs with strong experimental and computational evidence of MP-encoding potential (**∼**13% of the initial MP collection). Using The Cancer Genome Atlas (TCGA), we identified 2606 high-confidence lncRNA-derived MPs encoded by 647 genes across 16 cancer types. We then focused on 501 MPs from 124 lncRNA genes whose expression changes significantly across tumor stages and metastatic transitions, representing cancer transitional lncRNAs (Tr-lncRNAs). Dipeptide composition and conservation analyses showed that these MPs differ from a size-matched human coding proteome, supporting their potential as neoantigens. All possible 9-mer peptides were evaluated for predicted binding to prevalent European HLA class I alleles. Approximately 60% of Tr-lncRNA genes and 184 (37%) of derived peptides exhibited strong predicted HLA binding. Peptides from XIST, PCAT7, PVT1, HAND2-AS1 showed broad HLA coverage. Notably, TTN-AS1, encoded an MP (79 aa) generated 33 predicted distinct epitopes spanning all 27 HLA alleles. Our analysis identifies lncRNA-derived MPs as a previously underexplored source of potential cancer neoantigens, highlighting their promise as biomarkers and targets for immunotherapy.

**Availability:**

Data, code and supplementary materials are available in https://doi.org/10.5281/zenodo.20167452 and GitHub: https://github.com/stavzok1/lncrna_peptide_analysis.

## 1 Introduction

Cataloging long non-coding RNAs (lncRNAs) has identified ∼36 000 genes and ∼190 000 transcripts in the human genome (Mudge *et al*. 2025). Although their transcriptional regulation resembles that of protein-coding mRNAs, lncRNAs possess distinct genomic and molecular features. Many are enriched in the nucleus and chromatin but are also detected in exosomes, and most show low expression levels combined with strong tissue and cell specificity (Mattick *et al*. 2023).

Recent advances in proteomics and translation profiling have revealed that many lncRNAs contain short open reading frames (sORFs) capable of encoding peptides of 10–100 amino acids (aa), termed micropeptides (MPs) (Choi *et al*. 2019). Ribosome profiling (Ribo-seq) and proteogenomic studies suggest that hundreds to thousands of such MPs may be produced, although experimentally validated examples remain limited (Tharakan and Sawa 2021, Prensner *et al*. 2023). Because direct experimental evidence is sparse, the study of lncRNA-derived peptides relies largely on computational and indirect experimental approaches (Xiao *et al*. 2024). Evolutionary approaches are often limited by the weak conservation of these short sequences (Ruiz-Orera *et al*. 2020), and translation may occur through noncanonical mechanisms such as internal ribosome entry sites (IRES), particularly under stress conditions (Liu and Qian 2014).

Direct detection methods including Ribo-seq and mass spectrometry (MS) provide partial evidence for translated sORFs, yet many predicted peptides appear unstable and are rapidly degraded (Slavoff *et al*. 2013). Computational frameworks integrating Ribo-seq data, such as PepScore, estimate the likelihood that translated sORFs produce stable peptides in human cells (Yang *et al*. 2024).

lncRNAs are increasingly recognized as key regulators of cancer biology, influencing chromatin remodeling, transcriptional programs, RNA-protein interactions, and post-transcriptional regulation. Their dysregulated expression is widespread across cancer types and often reflects lineage identity and tumor progression states (Prensner and Chinnaiyan 2011, Nieto *et al*. 2016). Beyond regulatory functions, lncRNA-derived MPs may expand the repertoire of tumor-derived proteins.

Importantly, tumor-derived peptides presented on HLA molecules constitute neoantigens that can trigger immune recognition. The presence or absence of such neoantigens contributes to the distinction between immunologically “hot” tumors that respond to immune checkpoint therapy and “cold” tumors that evade immune detection (Wang *et al*. 2023). Identifying new sources of neoantigens is therefore critical for improving cancer immunotherapy and understanding immune escape mechanisms.

In this study, we investigate the protein-coding potential of lncRNAs and their association with cancer progression and metastasis. Using differential expression analysis of TCGA transcriptomic data (Tomczak *et al*. 2015), we identify lncRNAs whose expression changes across tumor stages and metastatic transitions, termed transitional lncRNAs (Tr-lncRNAs), many of which are cancer-type specific (Zok *et al*. 2025). We systematically characterize lncRNAs with strong evidence of peptide-coding capacity and identify 2,606 high-confidence lncRNA-derived MPs. We further analyze a subset of transitional lncRNA-derived MPs to assess their sequence features and their potential to generate immunogenic peptides presented by HLA molecules. Our results suggest that lncRNA-derived MPs represent a previously underexplored layer of the tumor immunopeptidome with potential relevance for cancer progression and immunotherapy.

## 2 Methods

### 2.1 Naming and harmonization of lncRNAs

TCGA lncRNA genes were taken from GENCODE v23 gene annotations: expression matrix columns are gene symbols, and Ensembl gene IDs were matched to those symbols using full versioned ENSG or the unversioned stem, via the same v23-derived gene table. SmProt2 peptides were tied to TCGA when SmProt’s symbol matched a matrix column or when SmProt’s GeneID mapped to a matrix symbol through that table. Restriction to Tr-lncRNAs used the same symbol/ID logic on the limma–z gene list. Peptide amino-acid (aa) sequences were rebuilt from SmProt’s transcript coordinates by slicing the corresponding GENCODE v49 spliced transcript and translating.

#### 2.1.1 Overall filtering scheme and terminology

To define the background lncRNA universe from which transitional lncRNAs (Tr-lncRNAs) were identified, we used curated RNA-seq data from The Cancer Genome Atlas (TCGA), accessed through Xena’s RNAseq–TOIL RSEM dataset (Tomczak *et al*. 2015). From the TCGA-represented transcriptome, we extracted an inclusive set of 14 855 lncRNA genes. Tr-lncRNAs were then defined through a staged filtering strategy. First, RNA-seq samples were filtered to exclude healthy or non-primary tumor samples, resulting in 9182 tumor samples across 32 cancer types.

These samples were subsequently stratified according to pathological annotations. For analyses based on pathologically defined tumor stage, 6256 samples had available staging information. After retaining only cancer types with more than 100 samples, this dataset comprised 5640 samples from 13 cancer types. In parallel, for analyses based on disease progression from primary to metastatic disease, 5323 samples had relevant annotations. After applying the same cancer-type sample-size threshold, this dataset comprised 4854 samples from 15 cancer types. Combining the cancer types from the metastasis and stage analyses resulted in 16 cancer types.

#### 2.1.2 Clinical data for major cancer samples

Clinical metadata, including cancer type, tumor stage, and survival outcomes, were curated from the Pan-Cancer Atlas of TCGA, ensuring consistent stage classification across studies. We used pathological stage annotations and collapsed sub-stages into major stages (e.g. IIA/IIB → II). Metastasis status (M) was derived from TNM annotations: M0 indicated non-metastatic (local/regional) tumors and M1 indicated distant metastasis. Among M0 tumors, we further stratified cases by T stage to capture differences in primary tumor size/extent.

### 2.2 Differentially expressed transitional lncRNAs

RNA sequencing (RNA-seq) data and associated clinical annotations were obtained from TCGA via the UCSC Xena portal (Tomczak *et al*. 2015). Raw read counts for annotated lncRNAs were extracted, processed, and normalized (Vivian *et al*. 2016). Expression was reported as RSEM (RNA-seq by expectation maximization) and log2[expected_counts + 1]-transformed to mitigate bias from low counts. Cancer types with less than 100 samples were omitted.

Clinical annotations included pathological stage, T stage, and M state. For each transition (e.g. I→II), lncRNAs with expected counts ≥1.0 in ≥40% of samples (both stages combined) were retained. Within cancer and transition differences in log2 fold change (FC) of mean expression were standardized to z-scores, after confirming approximate normality (see Supplementary data in GitHub). Transitions included I→II, II→III, III→IV, and a combined early-to-late (E→L) category. Metastasis-related groupings were: (1) non-metastatic tumors stratified by primary tumor size (M0-small: T1 ≤ 2cm; M0-large: T2–T4 > 2cm), (2) M0-large versus metastatic tumors (M1), and (3) a simplified early (ME) versus late (ML) progression.

The limma tool (ver. 3.66.0) was used with a two-group design (early vs late disease stage) per cancer type and predefined transition. Models used moderated t-statistics with Benjamini–Hochberg FDR <0.05 for significance, contrast with fewer than 10 samples per group were omitted. lncRNA genes with *|z|* ≥ 3 in any stratum and which resulted significant in any limma contrast (union over stage and metastasis analyses at FDR <0.05) were designated Tr-lncRNA. This intersection, across the 7 transitions and 16 cancer types, resulted in 1608 Tr-lncRNAs.

### 2.3 Database of micropeptides (MPs) and epitope mapping

To perform microprotein (MP) analysis, we downloaded the SmProt database (Li *et al*. 2021) and filtered MPs by requiring both translation initiation site (TIS) *P*value and ribosome footprint enrichment considering periodicity (Ribo-seq). Recall that the periodic behavior is an informative feature in the algorithms used for reliability of translated open reading frames (ORFs). We demand that both measurements will meet *P* value of <0.05. This filtering resulted in 2606 MPs, referred to as lncRNA-MPs, originating from 647 unique lncRNA genes. Among these, 124 lncRNA genes overlapped with the Tr-lncRNA set and collectively encoded 501 MPs, which we termed Tr-lncRNA-MPs.

We used the Allele Frequency Net Database (AFND) to examine population frequencies of immune-related loci, including HLA. The data contains allele, haplotype, and genotype information from large population origins covering 14 M individuals worldwide (Gonzalez-Galarza *et al*. 2020).

### 2.4 NetMHCpan-4.2, IEDB class I next-generation analysis, and strong binders

Nonamer epitopes were generated with a sliding window (step 1) over each MP and scored with NetMHCpan-4.2 in 9-mer mode across a fixed 27-locus HLA class I panel. The same peptides were submitted to the IEDB MHC Class I next-generation pipeline (IEDB Tools API, mhci tool group). That run combined two predictor heads: (i) processing with method basic processing, using the immunoproteasome model, TAP transport with IEDB defaults (TAP precursor length 1, TAP α = 0.2), and NetMHCpan4.1 BA as the internal peptide–MHC affinity model inside the pathway; (ii) class I immunogenicity with custom anchor masking at residue positions 2, 5, and 9 (9-mers). For downstream “strong binder” (SB) calls we required, on each peptide–allele row, IEDB immunogenicity score >0.1, IEDB processing score >1.5, NetMHCpan-4.2 EL % rank <1%, and predicted MHC-I IC50 < 150 nM as reported from the IEDB NetMHCpan-4.1 binding output which were consistently stricter than the NetMHCpan-4.2 binding affinities.

When counting SB epitopes derived from an MP, two counting methods were used: (i) Unique, each epitope was counted only once. (ii) Instance, the epitope was counted separately for each HLA to which it was identified as an SB.

### 2.5 Coding proteome sampling (whole proteins)

For a control matched to short MPs, we sampled the entire annotated human coding proteins (UniProt reference proteome) to match the size distribution of the analyzed MPs. Parent proteins were grouped by sequence length bins with 5aa width, spanning the MPs length range; within each bin we drew whole proteins so that the length-bin mixture of the control cohort matched the mixture observed in the lncRNA MP parent set as closely as possible for a fixed total number of control parents. Where the pool in a bin was still larger than the bin’s initial allocation, an additional top-up draw from under-filled bins was applied until the target cohort size was reached or pools were exhausted.

### 2.6 t-SNE dimensionality reduction

We applied t-SNE (t-distributed stochastic neighbor embedding) using scikit-learn 1.8.0 to visualize the expression data. Each point represents a tumor sample. Using the sklearn t-SNE package, samples were projected into 2D space from a data frame of rows (samples) and columns (lncRNA expressions). Spatial proximity reflects overall similarity in lncRNA expression profiles.

### 2.7 Conservation labeling

Human lncRNA conservation was assessed using genome alignments across 40 vertebrates (zebrafish to human). Homologous sequences required ≥50 nt and >20% of the lncRNA length. LncBook (Li *et al.* 2023) reports 22,347 human lncRNAs with some homology. For each species, sequences were considered homologous if alignment length and identity exceeded the intronic Q50 threshold. Conservation at Q75 indicates an exon or intron score higher than 75% of comparable lncRNAs.

### 2.8 Data availability and Python libraries

Visualizations and large data tables are available on https://doi.org/10.5281/zenodo.20167452. GitHub includes code, figures and supplementary materials (https://github.com/stavzok1/lncrna_peptide_analysis). The following software was used. Python: (i) Matplotlib 3.10.8 for data visualization; (ii) NumPy 2.3.4 and pandas 2.3.3 for numerical and tabular data handling; (iii) SciPy 1.16.3 (scipy.stats) for hypothesis tests (Fisher’s exact test, *χ*^2^ tests, Spearman correlation, Mann–Whitney U); (iv) scikit-learn 1.8.0 for sample embedding (t-SNE, PCA) and scaling; (v) Biopython 1.86 for FASTA sequence I/O in the micropeptide and NetMHC workflows; (vi) logomaker 0.8.7 for sequence logos in Fig. 6; (vii) openTSNE 1.0.4 for supplement sample embedding (t-SNE). Benjamini–Hochberg false discovery rate (FDR) adjustment for amino-acid and dipeptide composition tests was implemented in Python (NumPy 2.3.4). R: differential expression of lncRNAs and BH-FDR on adjusted *P* value used limma 3.66.0 (Bioconductor) and jsonlite 2.0.0.

## 3 Results

### 3.1. Classification of major cancer types by lncRNA expression profiles

**Figure 1:**
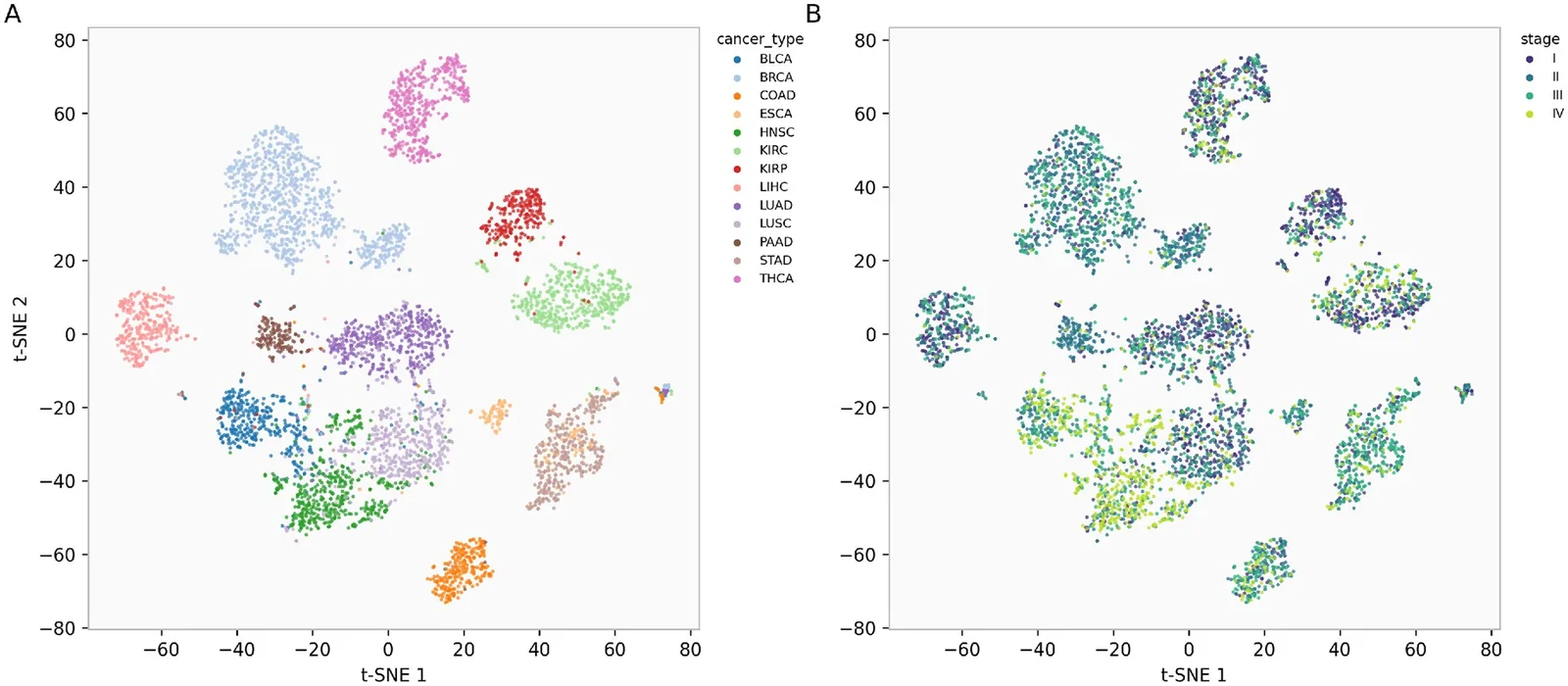
Cancer type clustering by t-SNE by lncRNAs expression profiles. (A) Each data point represents different sample. The color coded for the 2D-embedding is shown for the cancer types (total 13) from primary tumors in TCGA. (B). Same 2D embedding from t-SNE as in (A). However, the color labels are according to the four pathological stage.

From a collection of ∼190,000 lncRNA-associated transcripts (GENCODE v49), we asked whether the presence of expressed lncRNAs as the sole information from isolated samples can signify cancer types with high precision. We performed clustering by reducing dimensionality using 2D visualization t-SNE (see Methods) with all lncRNAs reported for each sample according to their cancer types (Fig. 1A).

The t-SNE analysis validated that lncRNAs capture information that supports a successful classification of all major cancer types. We tested the possibility that the successful partition of the cancer types is dominated by their pathological stage. To this end, we colored the samples by their matched stage labels (I to IV, Fig. 1B). While it is evident that some preferences were observed for specific cancer types (e.g. stage IV dominating HNSC), for most cancer types, the mixing of all stages confirmed that it is not the stage data that supports cancer type classification. The information captured in PC3 and PC4 corroborated the results (see Supplementary data).

### 3.2 Cancer transitional lncRNAs (Tr-lncRNAs) were detected across 16 major cancer types

We focused on a small subset of lncRNAs that are likely to function through their encoded MPs in cancer samples from major cancer types in TCGA. We compiled a set of 2606 MPs derived from 647 lncRNA genes (lncRNA-MPs). Notably, this set represents approximately 13.1% of the unfiltered collection of potential peptides (19 920).

**Figure 2:**
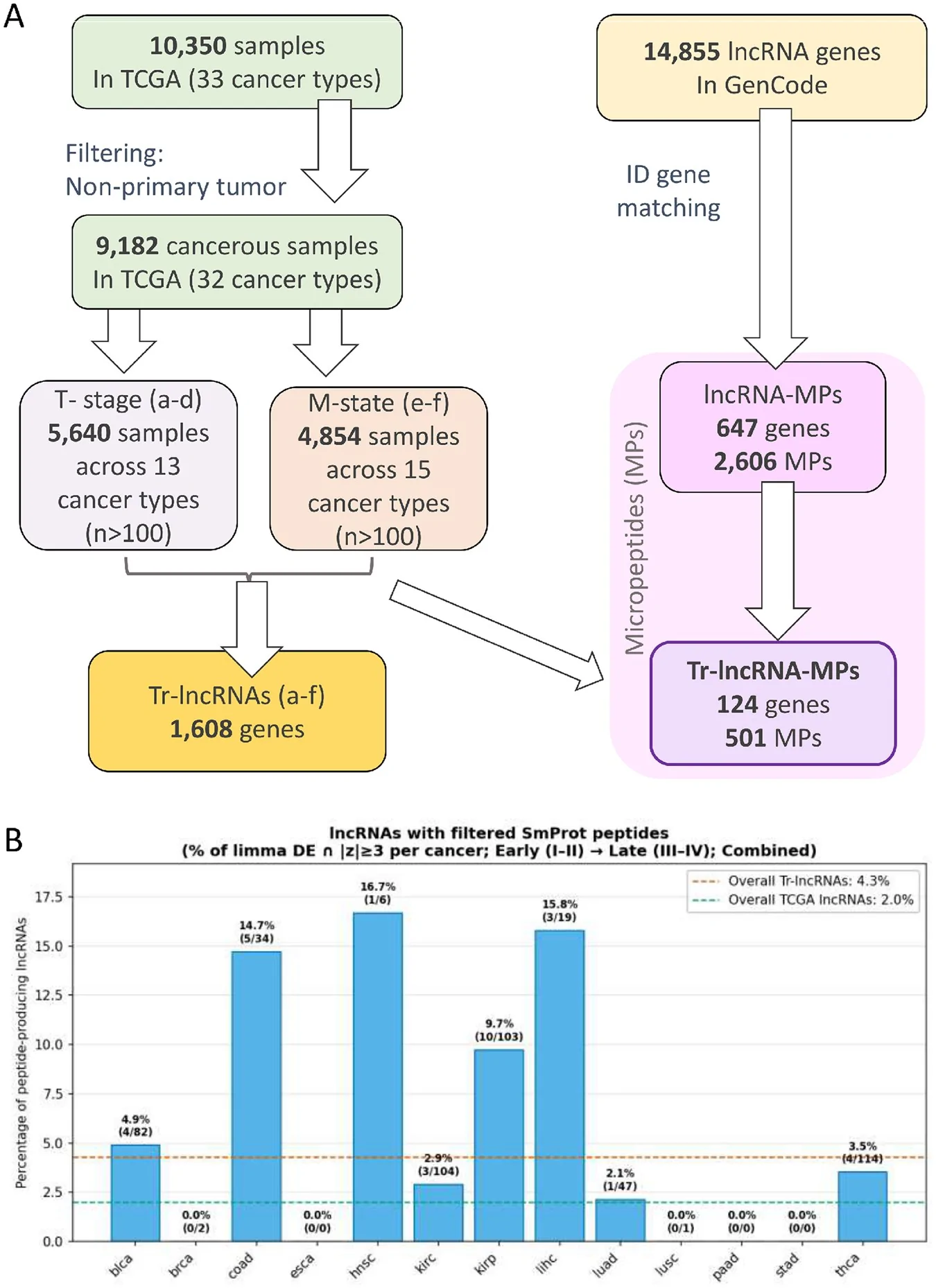
Percentage of lncRNAs producing MPs among Tr-lncRNAs across cancer types. (A) The filtration scheme for lncRNAs in TCGA and the subset of Tr-lncRNAs that produce high quality MPs. (B) The fraction of Tr-lncRNAs producing high confidence MPs in Early->Late transition *(*E→L, T-stage) across major cancer types, labelled and abbreviated as in TCGA. The dashed green and red horizontal lines represent the proportion of lncRNAs producing confident MPs among all lncRNAs and among the Tr-lncRNAs, respectively.

Each lncRNA was analyzed for differential expression across consecutive stages, partitioned into up- and downregulation patterns, and across major cancer types (Fig. 2A). We observed numerous lncRNAs associated with specific cancer types and distinct transition states. LncRNAs that were differentially expressed (*|z|* > 3, limma *P* < 0.05) and showed consistent behavior across cancer samples were termed transitional lncRNAs (Tr-lncRNAs) (Zok *et al*. 2025). Tumor transitions based on pathological stages (4) and metastasis status (3), yield a total of seven transitions that collectively identified 1,608 Tr-lncRNAs (Fig. 2A).

### 3.3 A Small fraction of Tr-lncRNAs encode MPs

We focus on the protein capacity from lncRNAs (lncRNA-MPs), and specifically among them, the subset of transition-significant (Tr-lncRNAs). We further filtered out Tr-lncRNAs across major cancer types by demanding MP-production capacity. To this end, we applied a confidence threshold using the internal statistics of the SmProt scoring. The SmProt considers high confidence MPs by matching with Translation initiation sites (TIS) and high confidence Ribo-seq experimental results (see Methods).

Altogether for any of the 7 transitions, cancer types (total 16) and expression directionality (up- and downregulation), we report on 647 lncRNAs that are the source of 2606 MPs (≥10 aa) among them, 501 Tr-lncRNA-MPs from 124 genes will be further discussed. Figure 2B shows that the overall frequency of high confidence Tr-lncRNA-MPs is significantly higher than the default occurrence of lncRNA-MPs in the lncRNA collection - 2.0% of TCGA lncRNAs produce MPs in general, versus 4.3% among the Tr-lncRNAs (Fig. 2B, compared the dashed green and red lines). We conclude that by restricting MPs to Tr-lncRNAs and to a specific cancer type, the most reliable lncRNA genes are exposed, along with strong evidence for their differential expression along cancer progression.

### 3.4 Dipeptide composition of Tr-lncRNAs-MPs differs from canonical proteins

**Figure 3:**
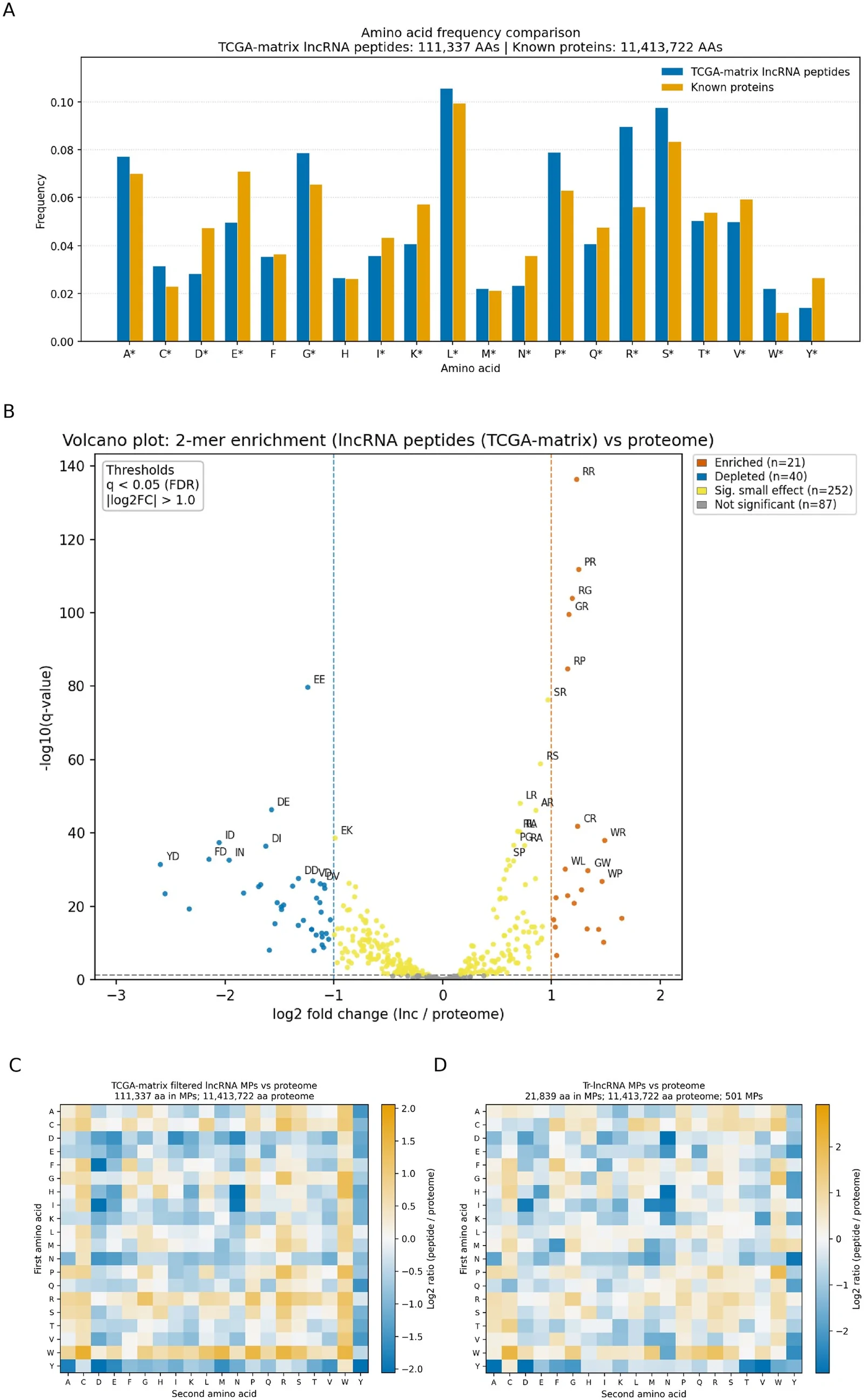
Amino acid (aa) composition among MPs. (A) Composition of aa in the human proteome and the lncRNA-MPs. Statistically significance difference is marked by asterix (Fisher’s exact test BH-FDR < 0.05). (B) Volcano plot of the dipeptide occurrences marking the statistically significant for the enriched (red) and depleted (blue) sets. (C) A matrix showing all 400 dipeptides pairs and their log2 ratio of lncRNA-MPs and human proteome. (D) The differential frequency of each dipeptide in the Tr-lncRNA-MPs (total 21.8k aa) relative to known human proteome (11.4M aa).

We tested whether the composition of aa in lncRNA-MPs is identical to that of the human proteome. We found that the lncRNA-MPs deviate substantially from the aa composition of coding genes. Out of the 20 aa, 18 were significantly different (Fig. 3A, asterisk). The difference in lncRNA-MPs relative to canonical proteins is also identified in their dipeptide compositions.

Figure 3B shows a Volcano plot of the dipeptide occurrences in lncRNA-MPs and the human proteome (11.4M aa) marking the statistically significant for the enriched (21, red) and depleted (40, blue) sets. Figure 3C is a heatmap by log_2_-transformed frequency ratios for dipeptide from all confident lncRNA-MPs relative to all canonical human proteins. The lncRNA-MPs (111k aa) show that dipeptides ID, YA, YD are strongly underrepresented while WC, WW dipeptides are overrepresented relative to human proteome. Figure 3D shows the significant peptide set of all 124 Tr-lncRNAs genes which produce peptides relative to human proteome. We found that numerous dipeptides (i.e. MF, HN, EY, DN, YT) are depleted. We concluded that the predicted ORFs in lncRNAs are not merely a random set of the human coding proteome.

### 3.5 Validation by evidence for Tr-lncRNA-MPs

Table 1 shows list of Tr-lncRNAs with multiple (at least 10) MPs. The gene length is PVT1, LINC00511 and LINC01515 are >300kb each. We show that for some of these genes, evolution evidence indicates coding genes as their homologues. For example, PVT1 coding homologs were found in marmoset, chimp, bonobo, orangutan and gorilla (Table 1). We consider 501 peptides that belong to 124 genes as peptide candidates along cancer progression.

**Table 1:** Tr-lncRNA with MPs (>10 per gene).

Gene symbol	Ensemble ID	n MPs	Size aa	n MPs (>30 aa)	^a^Con.
XIST	ENSG00000229807.12	30	96	20	0
PVT1	ENSG00000249859.11	27	90	17	5
LINC00511	ENSG00000227036.8	16	74	14	1
MIR7-3HG	ENSG00000176840.12	15	84	8	3
DUXAP8	ENSG00000206195.11	15	72	9	0
HAND2-AS1	ENSG00000237125.10	11	83	10	1
LINC01515	ENSG00000228065.11	11	34	1	0

a Evidence of protein-coding among conserved (Con) n homologues.

**Figure 4:**
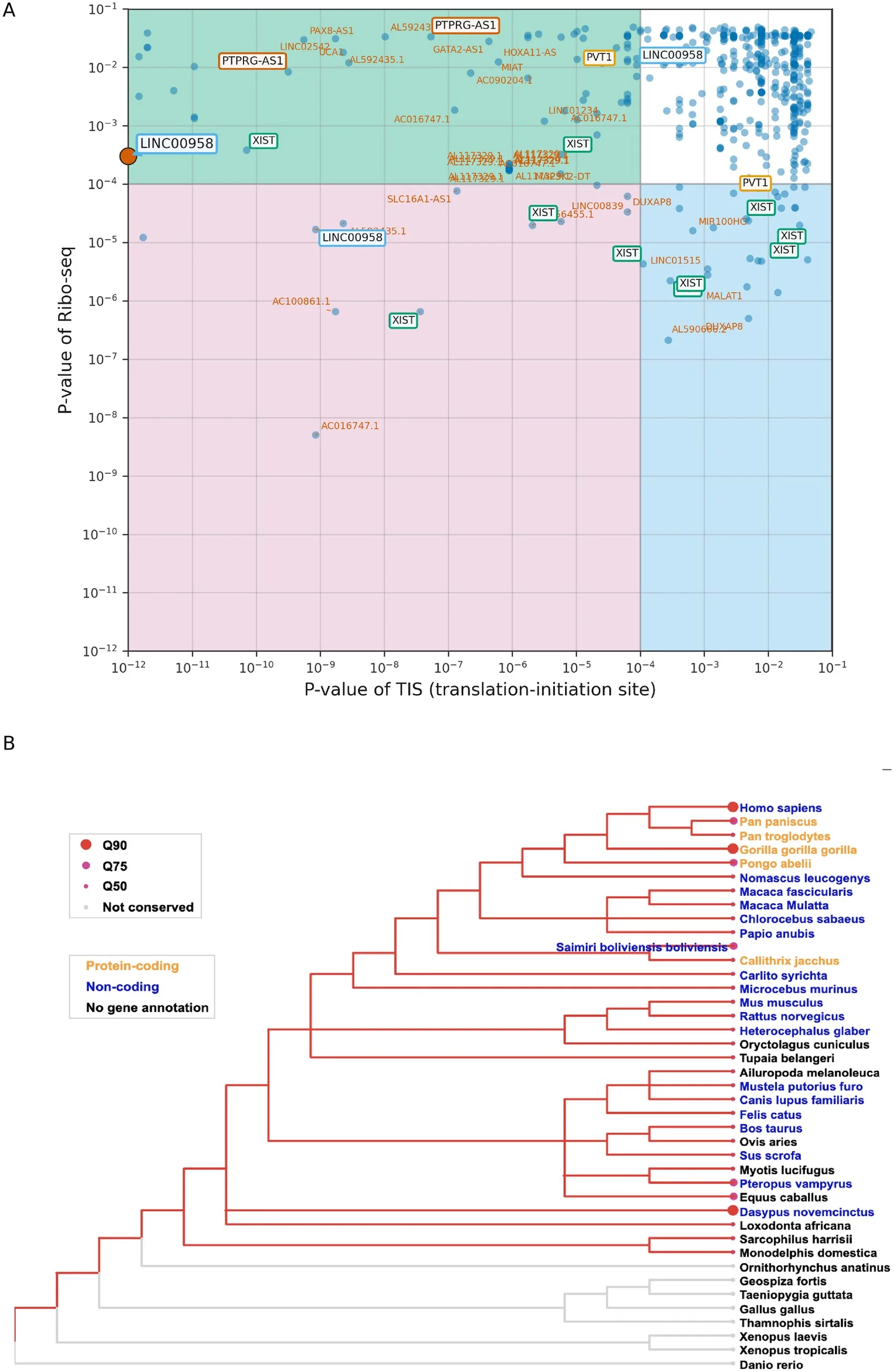
Characterization of Tr-lncRNA-MPs. (A) *P* value of TIS and Ribo-seq for Tr- ncRNA-MPs. Each dot marks specific MPs (total 501). All values are *P* ≤ 0.05. The shaded areas are MPs that are strongly supported by either TIS (green) or Ribosome profiling (light blue) *P* value (<1e-04). Frames with gene symbols mark representative genes with multiple labels. (B) Illustration for the conservation map for CDKN2B-AS1 gene for 40 species based on LncBook2. Red branches indicate the existing homologs. In blue font the noncoding genes, and in orange, the coding genes. Gray branches indicate species with no homologs to the human gene.

Figure 4A analyzes the strength of evidence collected for each of the proposed Tr-lncRNA-MPs. We compared two statistical measurements of TIS combined with ribosome profiling. All 501 MPs are with *P* value <0.05 in both measurements. A few of the 124 genes of Tr-lncRNA-MPs were very significant by both of the measurements (*P* value <1e-04 each). Among them are known (e.g. XIST), and poorly studied lncRNAs (e.g. LINC00958) (Fig. 4A).

We further tested the level of conservation and the evolutionary history of the discussed lncRNA-MPs. In CDKN2B-AS1 (Fig. 4B), the human gene has a coding gene as its close homologue in a related species (in two species of chimps). In this case, the homologs detected in all mammals (32 of 40 species), provide additional support for the coding capacity of this gene. We substantiated our conservation analysis by codon-based evidence using PhyloCSF (Phylogenetic codon substitution frequencies). The PhyloCSF uses a strong assumption that the codon-level conservation is consistent with known aa selection.

### 3.6 Tr-lncRNA-MPs as cancer epitope for MHC1

**Figure 5:**
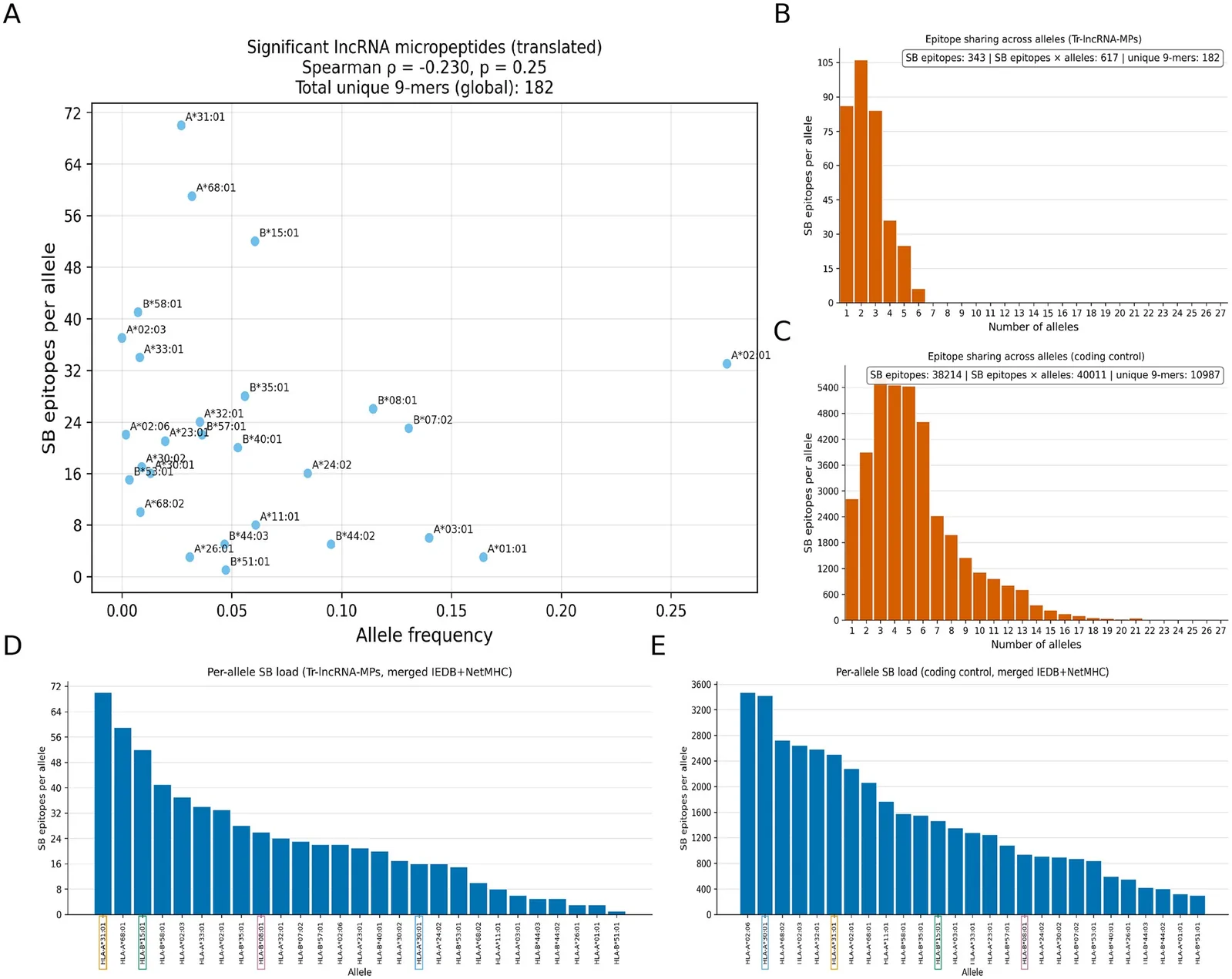
Tr-lncRNA-MPs in view of the MHC-I epitopes. (A) The 27 major HLA allele types that account for 98.5% of the European population. Epitope sharing across alleles: (B) The epitope occupancy for all Tr-lncRNA-MPs based on 343 SB epitopes. (C) The epitope occupancy derived from 500 short human proteins that are matched Tr-lncRNA-MPs size distribution. (D-E) 27 major human HLA alleles ranked by the occupancy of strong binders (SB) for Tr-lncRNA-MPs (D) and the human proteome as control. Color frames indicate cases of altered rank in occupancy for specific alleles.

We next asked whether Tr-lncRNA-MPs could generate effective MHC-I ligands. As a comparison, we selected a set of MPs from coding proteins that matched Tr-lncRNA-MPs length distribution. We focused on the potential epitopes that are encoded in each MPs. To this end, we performed in silico cleavage of all 501 MPs to 9-mer sequences (with moving window of a single aa). These 9-mer sequences were analyzed using NetMHCpan-4.2 and IEDB MHC Class I next-generation tools operated in a 9-mer mode and across 27 major HLA allele types that together account for 98.5% of the European population (see Methods). The frequency of the different alleles is estimated from the AFND database according to USA Caucasian population (Gonzalez-Galarza *et al*. 2020). For example, the HLA-A*02:01 covers 27.5% of that population (Fig. 5A).

Figure 5B shows that high-affinity 9-mers derived from Tr-lncRNA-MPs have a restricted allele occupancy, matching only 1 to 6 alleles (out of 27), with the highest frequency of epitopes shared by exactly two alleles. In contrast, size-matched peptides from the coding proteome demonstrate a much broader allele occupancy (Fig. 5C). While this control set also peaks at 2 to 3 shared alleles, it exhibits a long tail of epitopes capable of binding across a wide range of HLA alleles. For both analyses, we considered only strong binders (SB) according to the predicted affinity of the 9-mer to a specific HLA allele (see Methods). We conclude that high-affinity MHC-I 9-mer epitopes derived from Tr-lncRNA-MPs exhibit a highly restricted binding profile compared to the coding proteome, raising the possibility that they contribute a distinct, allele-specific subset of antigens to the cancer immunopeptidome.

An evaluation of the strong binder (SB) epitope load per HLA allele reveals that Tr-lncRNA-MPs and length-matched coding peptides exhibit distinct presentation preferences (Fig. 5D and E). For instance, HLA-A*31:01 displays the highest occupancy for Tr-lncRNA-MP 9-mers, yet it ranks as the 6th utilized allele for peptides derived from the coding proteome. Conversely, HLA-A*30:01 demonstrates the exact opposite trend, presenting a high volume of coding peptides but relatively few Tr-lncRNA-MPs (Fig. 5D and E).

### 3.7 Cancer MHC epitopes, the case of TTN-AS1

Our strict filters for Tr-lncRNA and the conservative evidence-based assignment of MPs resulted in 124 genes. TTN-AS1 was analyzed as an additional gene that is represented by an extreme number of potential MPs, with ∼1000 SB epitopes.

**Figure 6:**
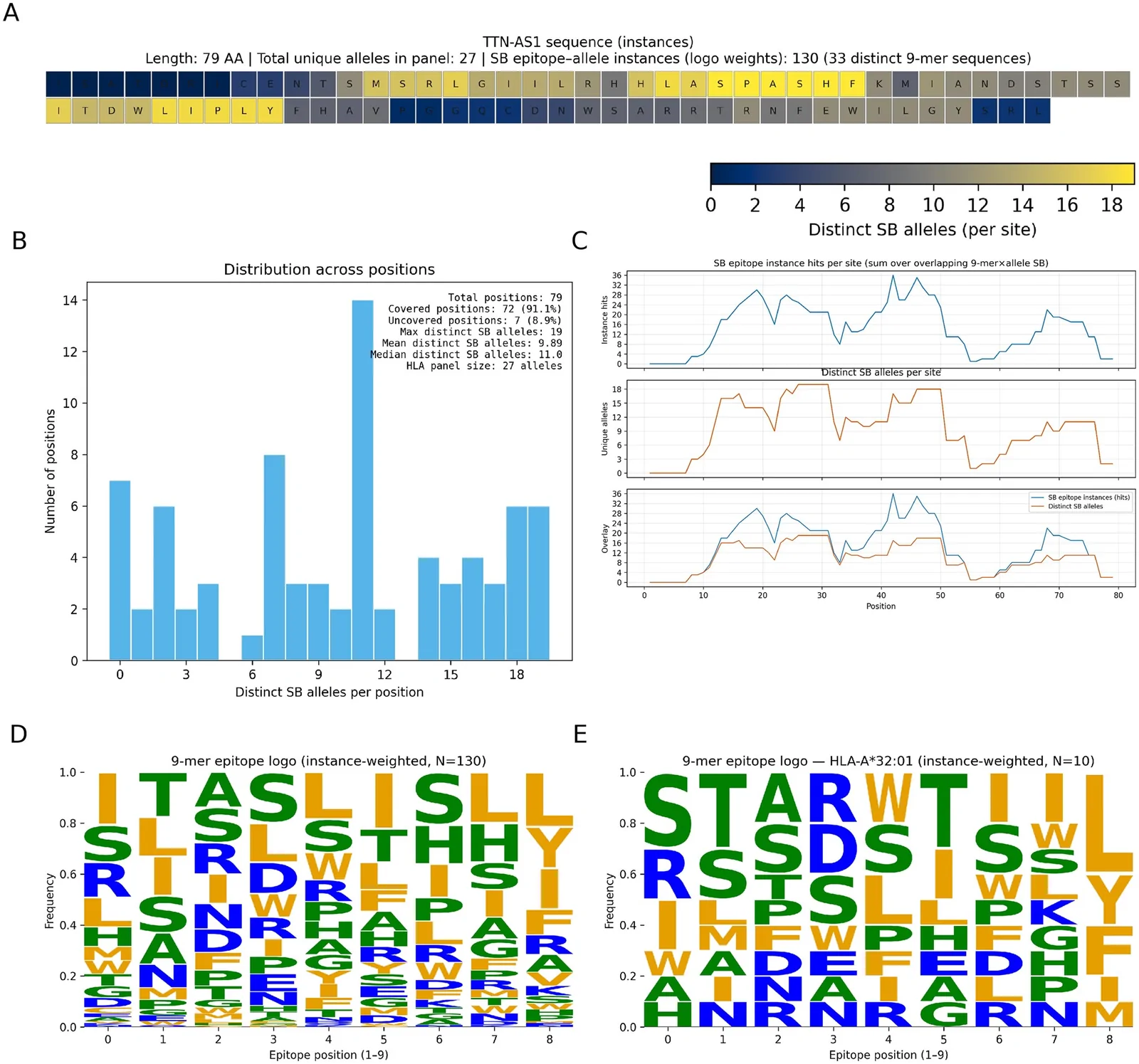
Characterization of TTN-AS1 by allele coverage. (A) A sequence of 79 aa was cleaved in-silico to 9-mer peptides and analyzed for the binding capacity. Allele coverage per residue is colored (yellow to blue). (B) Statistical analysis of the number of alleles across (79) positions. Maximal coverage per position is 19 HLA-alleles. (C) The epitope coverage (blue), allele diversity (red) across the sequence, and their overlap along the selected MP. (D) Logo presentation of the 9-mer along with the preference of aa along epitope position (aa 1–9) for all 130 sequences. (E) Example of a single HLA-A*32:01 with the 10 epitopes predicted to be strong binders (SB). The diversity of the 9-mer position is evident.

Analysis of the TTN-AS1-encoded MPs revealed a high density of predicted MHC-I epitopes with broad HLA allele coverage, indicating substantial immunogenic potential. Across the 79 aa sequence (Fig. 6A), multiple regions exhibited elevated epitope counts and allelic diversity (Fig. 6B and C), suggesting discrete immunogenic hotspots with potential population-wide relevance. Figure. 6D and E illustrate the diversity in binding, and the dominant 9-mer epitopes. The patterns at key anchor positions are consistent with canonical HLA-binding motifs and supportive of stable peptide–MHC interactions. Together, these findings indicate that the TTN-AS1 MPs include multiple high-confidence, allele-diverse epitopes and may contribute meaningfully to the tumor immunopeptidome.

## 4 Discussion

This study provides a pan-cancer framework for identifying MPs encoded by lncRNAs associated with tumor progression and metastasis. By integrating stage-resolved TCGA transcriptomics with Ribo-seq and translation initiation evidence, we prioritized 124 Tr-lncRNAs encoding 501 high-confidence MPs from a much larger candidate pool (2606 MPs). These peptides display distinct sequence composition relative to canonical proteins and are enriched for predicted MHC-I binding epitopes. Together, our results suggest that lncRNA-derived MPs represent a previously underexplored component of the tumor immunopeptidome and may contribute to progression-associated antigen landscapes.

Large-scale resources have greatly expanded the catalog of lncRNAs and non-canonical translation events. However, distinguishing functional peptides from background stochastic translation remains challenging. Several studies demonstrate that lncRNA-encoded MPs can have biological roles despite their limited length. For example, the lncRNA HOXB-AS3 encodes a conserved MP that modulates glucose metabolism and suppresses colon cancer growth (Wu *et al*. 2024). Proteogenomic analyses further show that peptides derived from lncRNAs and untranslated regions can be processed and presented by MHC class I molecules and recognized by T cells. Nevertheless, validated examples remain limited, highlighting the need for approaches that prioritize biologically relevant candidates. Our framework addresses this challenge by focusing on lncRNAs dynamically regulated during tumor progression and metastatic transitions.

We have shown in previous study that pan-cancer enrichment of Tr-lncRNAs supports the existence of conserved transcriptional programs accompanying tumor progression (Zok and Linial 2025). Here we show that a subset of these Tr-lncRNAs also encodes MPs and originates from well-characterized cancer-associated lncRNAs, suggesting that peptide production may represent an additional functional layer beyond the established RNA-mediated regulatory mechanisms (Prensner *et al*. 2023). These observations are consistent with emerging evidence that non-canonical translation products contribute to the repertoire of tumor-associated antigens (Laumont *et al*. 2018).

Recent immunopeptidomics pipelines combine large allele-diverse datasets with binding assays and MS evidence to improve prediction of clinically relevant epitopes (Pyke *et al*. 2023). Pan-cancer peptide atlas further indicate that non-canonical peptides can be presented at levels comparable to canonical coding genes across diverse tumors (Cai *et al*. 2025). Our results extend these observations by suggesting that lncRNA-derived MPs associated with cancer progression may represent a rich source of such antigens.

The immunological relevance of lncRNA-derived MPs is supported by studies demonstrating that non-canonical peptides can be processed and presented by MHC-I molecules and recognized by cytotoxic T cells (Chong *et al*. 2020). Because many lncRNAs show strong tissue and lineage specificity, their encoded peptides may generate antigens that are shared across tumors of similar origin rather than being strictly patient-specific (Mpakali and Stratikos 2021). Expanding the antigenic repertoire beyond canonical coding mutations is particularly relevant given the limited response rates observed for immune checkpoint blockade therapies (Sharma and Allison 2015). In this context, Tr-lncRNA-derived MPs may help explain part of the unexplored antigenic diversity present in tumors. Beyond immune recognition, the strong lineage and cell-state specificity of lncRNA expression suggests that lncRNA-encoded MPs may also serve as biomarkers reflecting tumor identity, plasticity, and metastatic potential (Nieto *et al*. 2016). Because Tr-lncRNAs are dynamically regulated across disease stages, peptides derived from these transcripts may provide molecular signatures of evolving tumor states and their immunogenic visibility.

Several limitations should be considered. First, lncRNAs are typically expressed at low levels, making differential expression analyses sensitive to sequencing depth, normalization, and cellular heterogeneity. Second, experimental validation of lncRNA-derived MPs remains technically challenging, as current MS approaches have limited sensitivity for low-abundance peptides within complex tumor proteomes (Slavoff *et al*. 2013). Third, we determine epitope as SB by combining different aspects that govern immunogenetic capacity (e.g. processing, specificity, affinity and surface presentation). The underlying tools and databases used (e.g. IEDB, NetMHCpan-4.2) may be biased towards the canonical proteome. Notably, HLA presentation pathways are frequently altered in cancer, which may prevent immunogenic peptides from reaching the cell surface (Leclerc *et al*. 2019). Consequently, predicted HLA binding does not necessarily translate into effective immune recognition.

This integrative statistical framework reveals a previously unrecognized layer of tumor-associated peptides encoded by lncRNAs, demonstrating that progression-associated transcriptional programs can give rise to a distinct class of candidate neoantigens beyond the canonical coding genome. Future work should integrate large-scale immunopeptidomics, targeted proteomics, and Ribo-seq to experimentally validate lncRNA-derived MPs and their presentation on HLA molecules. Such integrative approaches could help determine whether progression-associated lncRNA-derived peptides represent viable biomarkers or therapeutic targets, and may ultimately expand the repertoire of neoantigens available for cancer immunotherapy.
